# Acute Symptoms in Firefighters who Participated in Collection Work after the Community Hydrogen Fluoride Spill Accident

**DOI:** 10.1186/2052-4374-25-36

**Published:** 2013-11-28

**Authors:** Seong-Yong Cho, Kuck-Hyun Woo, Jin-Seok Kim, Seong-Yong Yoon, Joo-Yong Na, Jin-Hyun Yu, Yong-Bae Kim

**Affiliations:** 1 Department of Occupational and Environmental Medicine, Soonchunhyang University Gumi Hospital, 179, Gongdan 1-dong, Gumi-si, Gyeongbuk 730-706, South Korea; 2 Department of Preventive Medicine, Soonchunhyang University School of Medicine, Cheonan, 23-20, Bongmyung-dong, Cheonan-si, Choongchungnam-do 330-721, South Korea

**Keywords:** Hydrofluoric acid, Chemical hazard release, Firefighters

## Abstract

**Objectives:**

This study aimed to analyze the relationship between clinical status and work characteristics of firefighters and other public officers who engaged on collection duties in the site of the hydrogen fluoride spill that occurred on September 27, 2012, in Gumi City, South Korea.

**Methods:**

We investigated the clinical status, personal history, and work characteristics of the study subjects and performed physical examination and several clinical examinations, including chest radiography, echocardiography, pulmonary function test, and blood testing in 348 firefighters, police officers, volunteer firefighters, and special warfare reserved force who worked at the hydrogen fluoride spill area.

**Results:**

The subjects who worked near the accident site more frequently experienced eye symptoms (p = 0.026), cough (p = 0.017), and headache (p = 0.003) than the subjects who worked farther from the accident site. The longer the working hours at the accident area, the more frequently the subjects experienced pulmonary (p = 0.027), sputum (p = 0.043), and vomiting symptoms (p = 0.003). The subjects who did not wear respiratory protective devices more frequently experienced dyspnea than those who wore respiratory protective devices (p = 0.013). In the pulmonary function test, the subjects who worked near the accident site had a higher decease in forced vital capacity than the subjects who worked farther from the site (p = 0.019); however, no statistical association was found between serum calcium/phosphate level, echocardiography result, chest radiographic result, and probation work characteristics.

**Conclusions:**

The subjects who worked near the site of the hydrogen fluoride spill, worked for an extended period, or worked without wearing respiratory protective devices more frequently experienced upper/lower respiratory, gastrointestinal, and neurological symptoms. Further follow-up examination is needed for the workers who were exposed to hydrogen fluoride during their collection duties in the chemical plant in Gumi City.

## Introduction

Hydrogen fluoride is a colorless, potent respiratory irritant with an unpleasant odor. Exposure to hydrogen fluoride causes strong irritation in the eyes, nose, and throat and may result in tears, eye redness, rhinorrhea, sore throat, cough, headache, dermatalgia, and other symptoms. Even in cases in which no symptoms are present for 1–2 days after exposure, fever, cough, dyspnea, cyanosis, and pulmonary edema may occur later. If hydrogen fluoride or its aqueous solution, hydrofluoric acid, comes in contact with the skin, it causes serious tissue damage. Absorbing a considerable amount percutaneously or through the airway causes hypocalcemia and hyperkalemia, which leads to arrhythmia and may result in death. Chronic repeated exposure to hydrogen fluoride increases bone density, which is also known as fluorosis of the bones [1-3]. Hydrogen fluoride is used in etching and polishing glass, etching of silicon plates during the manufacture of semiconductors, as a catalytic agent for high-octane gasoline and in solutions that remove sand from metal moulds, and in the production of fluorine and aluminum fluoride, uranium purification, etc. [1-4].

At approximately 3:40 pm on September 27, 2012, a spill accident occurred at the National Industrial Complex chemical plant, where only hydrofluoric acid is produced. Approximately 8 tons of 100% hydrogen fluoride (anhydrous hydrofluoric acid) was spilled from inside the tank lorry. The accident caused the death of 5 workers who were working at the accident site. The total number of people who visited medical institutions and clinics as a result of the accident in the period until October 21, 2012, was 12,243, demonstrating that the accident had an extensive influence on the neighborhood residents [5-7].

Firefighters not only fulfill fire-extinguishing duties but also provide rescue and first-aid services during emergencies. In the process of performing such duties, they may be exposed to carbon monoxide, formaldehyde, hydrogen cyanide, hydrogen fluoride, and other combustion by-products [8-10]. Firefighters shut the gas release valve and attenuated the concentration of the hydrogen fluoride gas that was spilled around the accident site by sprinkling water [11]. One of the mass media reported that the number of firefighters and other public servants who needed medical help after performing collection duties after the accident was 414 and 90, respectively, altogether comprising 27.5% of the total 1,842 patients [12]. This suggests that the firefighters and other public servants who performed collection duties in the vicinity of the accident site were exposed to a large amount of hydrogen fluoride.

The literature on the health impact of hydrogen fluoride includes studies on acute and chronic health impact after a local community spill [3,7,13,14], an experimental study on the exposure of healthy males to low-concentration hydrogen fluoride [15], cases of skin burns [16-20], and cases of inhalation damage [21]. However, no literature was found on the health impact on firefighters and police officers who participated in collection after the hydrogen fluoride spill accident.

This study aimed to investigate the work history and clinical symptoms of the people who worked at the accident site as collectors of the spilled hydrogen fluoride and to determine the relationship between the distance from the accident site, working time, and use of protective devices, and the detrimental effects of hydrogen fluoride. The study will provide baseline information about the health management of personnel who participated in the hydrogen fluoride collection.

## Materials and methods

The subjects of this study were firefighters, police officers, volunteer firefighters, and special warfare reserved forces who were admitted to the general hospital in Gumi City for evaluation of the health impact of the hydrogen fluoride spill after performing hydrogen fluoride collection after the accident that happened on September 27, 2012, in the chemical plant in Gumi City. The total number of personnel who visited hospital in the period between October 1 and October 19, 2012, was 348 people, of whom 245 (70.4%) were firefighters, 46 (13.2%) were police officers, 34 (9.8%) were volunteer firefighters, and 23 (6.6%) were special warfare reserved forces.

### Study methods

A preliminary self-administrated survey and medical examination by interview were performed to determine the work history such as distance from the accident site, working time, and use of protective devices, as well as symptoms that appeared after performing duties at the accident site, physical examination, and personal history. When completing the survey, prior consent was obtained using a consent form for the collection and use of personal information. During medical examination by interview with the permission of the patient, serum calcium and serum phosphate analyses, pulmonary function test, chest radiography, and echocardiography were performed. To determine the individual level of exposure to hydrogen fluoride, the distance from the accident site was classified into less than 100 m, from 100 m to 1 km, and more than 1 km [7]. The working time was classified into less than 1 hour, between 1 hour and 10 hours, and more than 10 hours. The use of respiratory protective devices was classified into not used at all, used a disposable or cotton mask, used a gas mask, or used a self-contained breathing apparatus (SCBA; further on SCBA).

### Statistical analysis

A chi-square test for trend was performed on the general sociodemographic characteristics, symptoms according to body part, and complaints about separate symptoms related to the distance from the accident site, working time, and the use of respiratory protective devices. To compare the results of the clinical tests with the distance to the accident site, an analysis of covariance (ANCOVA) was used, compensating for age, sex, smoking, hospitalization duration, working time, and use of protective devices. The SPSS for Windows version 14.0 (SPSS Inc., USA) was used in the statistical analysis.

## Results

### Comparison between the general characteristics and symptoms according to distance from the accident site

The subjects were divided into those who stayed closer than 100 m, between 100 m and 1 km, and farther than 1 km from the accident site, and their characteristics were compared. Among the various age groups, 58.3% and 51.8% of those younger than 30 years and those 30–39 years old, respectively, worked closer than 100 m to the spill side, and 56.8% and 56.3% of those aged 40–49 years and those older than 50 years, respectively, worked between 100 m and 1 km from the site. With regard to sex, 42.1% of the men and 10.0% of the women worked closer than 100 m to the site; therefore, the men were more likely to work closer to the spill site than women. Of the firefighters, 47.3% worked closer than 100 m to the accident site, whereas most (56.5%, 67.6%, and 95.7%, respectively) of the police officers, volunteer firefighters, and special warfare reserved forces worked within 100 m to 1 km from the site. The results of the medical examination by interview revealed that the subjects had abnormal eye symptoms in 30% of the cases if they worked within 100 m from the accident site, in 17.3% of cases if they worked between 100 m and 1 km, and in 20.0% of the cases if they worked farther than 1 km from the spill site. Therefore, the subjects who worked the closest to the spill site had the most number of symptoms related to the eyes (p < 0.05). The other symptoms had no statistical differences depending on the distance from the accident site (Table 1).

**Table 1:** Comparison between the general characteristics and symptoms according to distance from the accident site

	**Less than 100 m (N = 140)**		**100 m-1 Km (N = 173)**		**More than 1 Km (N = 35)**		p-value*
	**N**	**%**	**N**	**%**	**N**	**%**	
**Age (years)**							0.000
**< 30**	21	58.3	14	38.9	1	2.8	
**30 ~ 39**	56	51.4	44	40.4	9	8.3	
**40 ~ 49**	45	32.4	79	56.8	15	10.8	
**≥ 50**	18	28.1	36	56.3	10	15.6	
**Gender**							0.031
**Male**	138	42.1	157	47.9	33	10.1	
**Female**	2	10.0	16	9.2	2	5.7	
**Smoking**							0.532
**No smoker**	100	42.2	112	47.3	25	10.5	
**Current smoker**	40	36.0	61	55.0	10	9.0	
**Job**							0.000
**Firefighter**	116	47.3	102	41.6	27	11.0	
**Police officer**	20	43.5	26	56.5	0	0.0	
**Volunteer firefighter**	4	11.8	23	67.6	7	20.6	
**Special warfare reserved force**	0	0.0	22	95.7	1	2.9	
**Abnormal symptoms**							
**Throat**^**†**^	24	17.1	36	20.8	4	11.4	0.082
**Nose**^**‡**^	18	12.9	17	9.8	6	17.1	0.924
**Eye**^**§**^	42	30.0	30	17.3	7	20.0	0.026
**Lung**^**∥**^	49	35.0	50	28.9	8	22.9	0.115
Skin**	15	10.7	20	11.6	3	8.6	0.886
**Heart**^**++**^	0	0.0	0	0.0	0	0.0	-
**Nerve**^**‡‡**^	15	10.7	14	8.1	1	2.9	0.141
**Stomach**^**‡‡‡**^	4	2.9	5	2.9	1	2.9	0.993

*Chi-square test for trend.

^†^Throat pain, change of voice, dry mouth.

^‡^Rhinorrhea, nasal obstruction, nasal pain, nasal bleeding.

^§^Ocular pain, red eye, blurred vision, lacrimination.

^∥^Cough, sputum, chest discomfort, dyspnea.

**Skin pruritus, skin rash, skin pain.

^++^Palpitation, left chest pain.

^‡‡^Fatigue, Headache, dizziness, arm/leg claudication, muscle weakness, inattention, muscle pain.

^‡‡‡^Vomiting, dyspepsia, nausea, diarrhea, abdominal pain.

### Comparison between the general characteristics and abnormal symptoms according to working duration at the accident area

The subjects were divided according to working time into those who worked less than 1 hour, those who worked between 1 and 10 hours, and those who worked for more than 10 hours, and their characteristics were compared. Working time, age, sex, and smoking status had no statistical differences. In all the groups of professions, most of the subjects worked between 1 and 10 hours, and 18.4% of the firefighters and 6.5% of the police officers worked for more than 10 hours, which was more than 0.0% volunteer firefighters and special warfare reserved forces. Longer hours of work was associated with more abnormal lung symptoms; that is, 10.7% of those who worked less than 1 hour, 31.6% of those who worked between 1 and 10 hours, and 37.5% of those who worked more than 10 hours had symptoms in the lungs. Symptoms in the gastrointestinal tract were observed in 0.0% of those who worked less than 1 hour, in 2.2% of those who worked between 1 and 10 hours, and in 13.8% of those who worked more than 10 hours. The longer the working time, the more subjects experienced symptoms in the gastrointestinal tract (p < 0.05). No statistically significant differences were observed for other body parts (Table 2).

**Table 2:** Comparison between the general characteristics and abnormal symptoms according to working duration at the accident area

	**Less than 1 hours (N = 28)**		**1-10 hours (N = 272)**		**More than 10 hours (N = 48)**		p-value*
	**N**	**%**	**N**	**%**	**N**	**%**	
**Age (years)**							0.972
**< 30**	1	2.8	30	83.3	5	13.9	
**30 ~ 39**	13	11.9	77	70.6	19	17.4	
**40 ~ 49**	11	7.9	114	82.0	14	10.1	
**≥ 50**	3	4.7	51	79.7	10	15.6	
**Gender**							0.569
**Male**	26	7.9	256	78.0	46	14.0	
**Female**	2	10.0	16	80.0	2	10.0	
**Smoking**							0.403
**No smoker**	17	7.2	186	78.5	34	14.3	
**Current smoker**	11	9.9	86	77.5	14	12.6	
**Job**							0.001
**Firefighter**	16	6.5	184	75.1	45	18.4	
**Police officer**	7	15.2	36	78.3	3	6.5	
**Volunteer firefighter**	4	11.8	30	88.2	0	0.0	
**Special warfare reserved force**	1	4.3	22	95.7	0	0.0	
**Abnormal symptoms**							
**Throat**^**†**^	8	28.6	45	16.5	22	22.9	0.840
**Nose**^**‡**^	3	10.7	30	11.0	8	16.7	0.344
**Eye**^**§**^	5	17.9	63	23.2	11	22.9	0.688
**Lung**^**∥**^	3	10.7	86	31.6	18	37.5	0.027
Skin**	2	7.1	32	11.8	4	8.3	0.946
**Heart**^**++**^	0	0.0	0	0.0	0	0.0	-
**Nerve**^**‡‡**^	1	3.6	22	8.1	7	14.6	0.079
**Stomach**^**‡‡‡**^	0	0.0	6	2.2	4	13.8	0.018

*Chi-square test for trend.

^†^Throat pain, change of voice, dry mouth.

^‡^Rhinorrhea, nasal obstruction, nasal pain, nasal bleeding.

^§^Ocular pain, red eye, blurred vision, lacrimination.

^∥^Cough, sputum, chest discomfort, dyspnea.

**Skin pruritus, skin rash, skin pain.

^++^Palpitation, left chest pain.

^‡‡^Fatigue, Headache, dizziness, arm/leg claudication, muscle weakness, inattention, muscle pain.

^‡‡‡^Vomiting, dyspepsia, nausea, diarrhea, abdominal pain.

### Comparison between the general characteristics and abnormal symptoms according to respiratory protector device used

The subjects were divided into those who did not use respiratory protective devices, those who used a disposable or cotton mask, those who used a gas mask, and those who used a SCBA, and their characteristics were compared. Of those younger than 30 years and those between 30 and 39 years of age, 58.3% and 47.7%, respectively, used SCBA. meanwhile, 33.8% of those aged 40–49 years used no respiratory protective device and 47.2% of those older than 50 years used a disposable or cotton mask. Of the men, 39.3% used a SCBA, whereas 50.0% of the women used no respiratory protective device. Compared with the other professions, the firefighters had the biggest percentage of those who used SCBA (53.9%). Of the police officers and special warfare reserved forces, 47.8% and 87.0%, respectively, used a disposable or cotton mask. Meanwhile, 61.8% of the special warfare forces used no respiratory protective device (p < 0.05). No other statistically significant differences were found in the symptoms by body part and the use of respiratory protective devices (Table 3).

**Table 3:** Comparison between the general characteristics and abnormal symptoms according to respiratory protector device used

	**No wear (N = 91)**		**Disposable or cotton mask (N = 114)**		**Gas mask (N = 11)**		**SCBA (N = 132)**		p-value*
	**N**	**%**	**N**	**%**	**N**	**%**	**N**	**%**	
**Age (years)**									0.000
**< 30**	5	13.9	8	22.2	2	5.6	21	58.3	
**30 ~ 39**	22	20.2	33	30.3	2	1.8	52	47.7	
**40 ~ 49**	47	33.8	46	33.1	4	2.9	42	30.2	
**≥ 50**	17	26.6	27	42.2	3	4.7	17	26.6	
**Gender**									0.012
**Male**	81	24.7	108	32.9	10	3.0	129	39.3	
**Female**	10	50.0	6	30.0	1	5.0	3	15.0	
**Smoking**									0.101
**No smoker**	56	23.6	79	33.3	5	2.1	97	40.9	
**Current smoker**	35	31.5	35	31.5	6	5.4	35	31.5	
**Job**									0.000
**Firefighter**	51	20.8	59	24.1	3	1.2	132	53.9	
**Police officer**	16	34.8	22	47.8	8	17.4	0	0.0	
**Volunteer firefighter**	21	61.8	13	38.2	0	0.0	0	0.0	
**Special warfare reserved force**	3	13.0	20	87.0	0	0.0	0	0.0	
**Abnormal symptoms**									
**Throat**^**†**^	13	14.3	21	18.4	3	27.3	27	20.5	0.262
**Nose**^**‡**^	10	11.0	12	10.5	1	9.1	18	13.6	0.468
**Eye**^**§**^	21	23.1	19	16.7	2	18.2	37	28.0	0.139
**Lung**^**∥**^	34	37.4	33	28.9	4	36.4	36	27.3	0.208
Skin**	5	5.5	19	16.7	1	9.1	13	9.8	0.977
**Heart**^**++**^	0	0.0	0	0.0	0	0.0	0	0.0	-
**Nerve**^**‡‡**^	9	9.9	8	7.0	0	0.0	13	9.8	0.840
**Stomach**^**‡‡‡**^	0	0.0	7	6.1	0	0.0	3	2.3	0.941

*Chi-square test for trend.

^†^Throat pain, change of voice, dry mouth.

^‡^Rhinorrhea, nasal obstruction, nasal pain, nasal bleeding.

^§^Ocular pain, red eye, blurred vision, lacrimination.

^∥^Cough, sputum, chest discomfort, dyspnea.

**Skin pruritus, skin rash, skin pain.

^++^Palpitation, left chest pain.

^‡‡^Fatigue, Headache, dizziness, arm/leg claudication, muscle weakness, inattention, muscle pain.

^‡‡‡^Vomiting, dyspepsia, nausea, diarrhea, abdominal pain.

### Analysis of the individual symptoms according to distance from the accident site

The results of the analysis of the manifestation of the individual symptoms according to distance from the accident site showed that the closer to the accident site the personnel worked, the more likely they were to experience cough symptoms, with 14.4% of those who worked within 100 m, 13.8% of those who worked within 100 m to 1 km, and 5.9% of those who worked further than 1 km experiencing cough. Fatigue and headache were also more common among those who worked at a closer distance, with the following percentages: 17.7%, less than 100 m; 7.1%, 100 m to 1 km; and 0.0%, farther than 1 km; and 8.6%, less than 100 m; 5.2%, 100 m to 1 km; and 0.0%, farther than 1 km (p < 0.05). Other symptoms had no statistically significant differences depending on distance from site (Table 4).

**Table 4:** Analysis of the individual symptoms according to distance from the accident site

	**Less than 100 m (N = 140)**		**100 m-1 Km (N = 173)**		**More than 1 Km (N = 35)**		**Total**		p-value*
	**N**	**%**	**N**	**%**	**N**	**%**	**N**	**%**	
**Throat**									
**Throat pain**	24	10.8	36	17.1	4	11.8	64	13.7	0.882
**Nose**									
**Rhinorreha**	15	6.8	13	6.2	4	11.8	32	6.9	0.698
**Nasal obstruction**	5	2.3	2	1.0	2	5.9	9	1.9	0.881
**Nasal pain**	1	0.5	2	1.0	0	0.0	3	0.6	0.932
**Eye**									
**Ocular pain**	28	12.6	22	10.5	5	14.7	55	11.8	0.143
**Red eye**	7	3.2	5	2.4	0	0.0	12	2.6	0.122
**Blurred vision**	4	1.8	4	1.9	2	5.9	10	2.1	0.612
**Lacrimination**	6	2.7	3	1.4	0	0.0	9	1.9	0.084
**Lung**									
**Cough**	32	14.4	29	13.8	2	5.9	63	13.5	0.017
**Sputum**	17	7.7	20	9.5	4	11.8	41	8.8	0.871
**Chest discomfort**	16	7.2	12	5.7	2	5.9	30	6.4	0.141
**Dyspnea**	1	0.5	1	0.5	2	5.9	4	0.9	0.084
**Skin**									
**Skin pruritus**	10	4.5	9	4.3	2	5.9	21	4.5	0.560
**Skin rash**	6	2.7	6	2.9	0	0.0	12	2.6	0.227
**Skin pain**	0	0.0	6	2.9	1	2.9	7	1.5	0.064
**Nerve**									
**Fatigue**	17	7.7	15	7.1	0	0.0	32	6.9	0.034
**Headache**	19	8.6	11	5.2	0	0.0	30	6.4	0.003
**Dizziness**	5	2.3	1	0.5	1	2.9	7	1.5	0.262
**Arm/leg claudication**	2	0.9	4	1.9	0	0.0	6	1.3	0.903
**Stomach**									
**Vomiting**	3	1.4	3	1.4	1	2.9	7	1.5	0.947
**Other**	4	1.8	6	2.9	2	5.9	12	2.6	0.459
**Total**	222	100.0	210	100.0	34	100.0	466	100.0	

*Chi-square test for trend.

### Analysis of the individual symptoms according to working time

The results of the assessment of the individual symptoms according to working time revealed that the longer the working time, the more frequent sputum was observed, with 0.0% of the workers who worked less than 0.0%, 9.5% of the workers who worked between 1 and 10 hours, and 9.6% of the workers who worked more than 10 hours experiencing sputum symptoms. The percentages for fatigue were 8.0% for less than 1 hour of work, 5.9% for 1–10 hours of work, and 10.8% for more than 10 hours of work, which was the highest percentage among those who worked over a long period. Vomiting was experienced by 0.0% of the subjects who worked less than 1 hour, by 0.8% of those who worked 1–10 hours, and by 4.8% of those who worked more than 10 hours, with more people experiencing the symptom as the working time increased (p < 0.05). Other symptoms had no statistically significant difference depending on working time (Table 5).

**Table 5:** Analysis of the individual symptoms according to working time

	**Less than 1 hours (N = 28)**		**1-10 hours (N = 272)**		**More than 10 hours (N = 48)**		**Total**		p-value*
	**N**	**%**	**N**	**%**	**N**	**%**	**N**	**%**	
**Throat**									
**Throat pain**	8	32.0	45	12.6	11	13.3	64	13.7	0.840
**Nose**									
**Rhinorreha**	2	8.0	23	6.4	7	8.4	32	6.9	0.207
**Nasal obstruction**	1	4.0	6	1.7	2	2.4	9	1.9	0.726
**Nasal pain**	0	0.0	3	0.8	0	0.0	3	0.6	0.830
**Eye**									
**Ocular pain**	4	16.0	45	12.6	6	7.2	55	11.8	0.713
**Red eye**	1	4.0	10	2.8	1	1.2	12	2.6	0.663
**Blurred vision**	0	0.0	7	2.0	3	3.6	10	2.1	0.094
**Lacrimination**	0	0.0	7	2.0	2	2.4	9	1.9	0.281
**Lung**									
**Cough**	2	8.0	49	13.7	12	14.5	63	13.5	0.056
**Sputum**	0	0.0	33	9.2	8	9.6	41	8.8	0.043
**Chest discomfort**	1	4.0	24	6.7	5	6.0	30	6.4	0.349
**Dyspnea**	0	0.0	4	1.1	0	0.0	4	0.9	0.803
**Skin**									
**Skin pruritus**	2	8.0	18	5.0	1	1.2	21	4.5	0.285
**Skin rash**	1	4.0	9	2.5	2	2.4	12	2.6	0.884
**Skin pain**	0	0.0	6	1.7	1	1.2	7	1.5	0.623
**Nerve**									
**Fatigue**	2	8.0	21	5.9	9	10.8	32	6.9	0.039
**Headache**	1	4.0	23	6.4	6	7.2	30	6.4	0.178
**Dizziness**	0	0.0	5	1.4	2	2.4	7	1.5	0.189
**Arm/leg claudication**	0	0.0	6	1.7	0	0.0	6	1.3	0.760
**Stomach**									
**Vomiting**	0	0.0	3	0.8	4	4.8	7	1.5	0.003
**Other**	0	0.0	11	3.1	1	1.2	12	2.6	0.844
**Total**	25	100.0	358	100.0	83	173.2	466	100.0	

*Chi-square test for trend.

The results of the assessment of the individual symptoms depending on whether the workers used a respiratory protective device indicated that dyspnea was observed in 4 people (4.4%) who did not use any protective device; however, it was not observed in those who used a cotton mask, a dust respirator, or a SCBA. Therefore, the proportion of the subjects who did not use a protective device was higher than that of the subjects who used protection (p < 0.05). Other symptoms did not have any statistically significant difference in terms of the use of respiratory protective devices (table not provided).

### Analysis of the results of the electrolyte analysis, pulmonary function test, electrocardiography, and chest radiography according to distance from the accident site

Serum calcium level, serum phosphorus concentration, and pulmonary function test results were analyzed according to the distance from the accident site. The mean serum calcium level was 9.41 mg/dL (range, 8.4-11.0 mg/dL). None of the subjects were calcium deficient, that is, a calcium level lower than 8.4 mg/dL. The results of pulmonary function test revealed that forced vital capacity (FVC) and FVC (%) were respectively 4.50 ± 0.67 L and 90.57 ± 10.01% for less than 100 m, 4.36 ± 0.75 L and 90.68 ± 11.35% for 100 m to 1 km, and 4.59 ± 0.86 L and 95.66 ± 11.43% for more than 1 km from the accident site, the latter being the highest values (p < 0.05). No other statistically significant difference was found based on the pulmonary function test results (Table 6).

**Table 6:** Analysis of the results of the electrolyte analysis, pulmonary function test, electrocardiography, and chest radiography according to distance from the accident site

**Characteristic**	**Less than 100 m (N = 140)**	**100 m-1 Km (N = 173)**	**More than 1 Km (N = 35)**	p-value*
	**Mean ± SD**	**Mean ± SD**	**Mean ± SD**	
**Serum electrolyte**				
**Calcium (mg/dL)**	9.41 ± 0.37	9.41 ± 0.37	9.45 ± 0.36	0.579
**Phosphorus (mg/dL)**	3.44 ± 0.70	3.44 ± 0.46	3.37 ± 0.35	0.767
**Spirometry**^**†**^				
**FVC (L)**	4.50 ± 0.67	4.36 ± 0.75	4.59 ± 0.86	0.019
**FVC% (%)**	90.57 ± 10.01	90.68 ± 11.35	95.66 ± 11.43	0.019
**FEV1 (L)**	3.67 ± 0.60	3.55 ± 0.62	3.64 ± 0.67	0.060
**FEV1% (%)**	91.80 ± 10.97	92.54 ± 11.61	95.60 ± 12.58	0.104
**FEV1/FVC**	81.63 ± 6.56	81.58 ± 6.02	79.45 ± 6.05	0.257
**FEF25-75 (L)**	3.70 ± 1.08	3.59 ± 0.97	3.40 ± 0.97	0.366
**FEF25-75% (%)**	95.20 ± 24.59	97.57 ± 23.85	94.80 ± 27.98	0.568

*Adjusted for age, sex, smoking, time of hospital visit, time of working at accident spot, and respirator protector using ANCOVA test.

^†^*FVC*, forced vital capacity; *FVC%*, FVC percent; *FEV1*, 1 second forced expiratory volume.

*FEV1*; FEV1 percent, *FEF25-75*, forced expiratory flow rate25-75; *FEF25-75%;* FEF25-75 percent.

Furthermore, no statistically significant differences were found in the results of the serum calcium, serum phosphorus, and pulmonary function tests depending on working time and the use of respiratory protection. On electrocardiography (abnormal findings in 29 people, 8.3%) and chest radiography (abnormal findings in 7 people, 2.0%), no statistically significant differences were found as well depending on the distance to the accident site, working time, and the use of protective devices (table not provided).

## Discussion

When performing their duties, firefighters may be exposed to various chemical substances that are the result of combustion. In particular, in case of accidents at industrial sites, chemical substances that are produced and used at the plant may have direct and indirect effects on the body in addition to the combustion gas that is released during the fire [9,22]. The hydrogen fluoride spill accident that happened on September 27, 2012, in Gumi City caused 5 deaths and resulted in a total number of 12,243 people visiting medical institutions and clinics before October 21, 2012. In addition, by November 6, 435 farming households experienced damage from the hydrogen fluoride spill, as well as 1,962 cars and 148 businesses. Not only neighborhood citizens but also agro-livestock products and plant equipment experienced extensive damage [5-7,23]. The primary air concentration of hydrogen fluoride measured at the time of the accident at 9.30 am on September 28, 2012, was 1 ppm. In the secondary examination performed at 2:30 pm, the concentration was undetectable [24]. However, if we consider that the hydrogen fluoride spill lasted for approximately 7 hours between the time of the accident at 3:40 pm on September 27, 2012, and the time when the valve was completely shut at 10:20 pm on the same day [11], the hydrogen fluoride concentration during the time when the firefighters and police officers were performing collection at the accident site may be assumed to be much higher than 1 ppm.

Hydrogen fluoride causes very strong irritations, including tears, throat tingling, nose irritation, cough, and headache [2,3,7,21]. The eyes are especially vulnerable to hydrogen fluoride exposure. Hydrogen fluoride may cause edema of the cornea and conjunctiva due to damage to the epithelial cells of the cornea and conjunctiva, which in severe cases may lead to loss of eyesight [25]. Many of the subjects in this study who worked within close range from the spill accident site also exhibited abnormal eye symptoms. Similarly, many of those who worked in close proximity to the accident site exhibited hydrogen fluoride irritation symptoms such as cough and headache. The above-mentioned irritation symptoms were estimated to be related to the fact that the closer the personnel were to the accident site, the higher the hydrogen fluoride concentration they were exposed to. As hydrogen fluoride is a gas with a very irritating smell and may cause strong neurological irritation, the workers were assumed to have experienced headaches frequently when they worked in close proximity to the accident site [7].

The subjects who performed work in the accident site for more than 10 hours exhibited symptoms in the lungs, including sputum. When high-concentration hydrogen fluoride is inhaled, it may immediately cause upper airway irritation, such as throat tingling and cough, and lower airway irritation, such as chest pain, dyspnea, sputum, pneumonia, and pulmonary edema. However, when low-concentration hydrogen fluoride is inhaled, it also first causes upper airway irritation and lower airway irritation may naturally follow [15,21]. In this study, we were not able to confirm the individual hydrogen fluoride exposure concentrations and the onset of sputum symptom. However, the subjects who performed collection duties for a long time may be assumed to have inhaled a greater amount of hydrogen fluoride than did those who worked for a short time. Therefore, among those who performed collection duties in the accident site for a long time, the percentage of the subjects who exhibited lower airway irritation was higher. In addition, vomiting, a gastrointestinal symptom, was observed frequently in the workers who worked for a long period. This may be attributed to the strong irritating smell of hydrogen fluoride [25], psychological stress, or the effect of hydrogen fluoride that is excreted into the digestive system by action of the cilium of the bronchi [7].

Firefighters use SCBA at fire-extinguishing and rescue sites to protect their respiratory organs. This equipment supplies the members of the fire brigade with oxygen and prevents them from inhaling smoke and combustion gas; it is approximately 200 times more effective for protecting the respiratory organs than a dust respirator that has an appropriate purification cartridge [9,26,27]. The US Centers for Disease Control and Prevention mention that during a hydrogen fluoride spill, wearing a SCBA and chemical protective suit is necessary [28]. The total number of workers who used respiratory organ protective devices in this study, including the 137 firefighters who wore air respirators, was 258. None of them experienced dyspnea, whereas of the 91 workers who did not use protection, 4 developed dyspnea (p = 0.013). The results of the pulmonary function test in the 4 workers showed that apart from the 1 worker who previously had asthma, the other 3 workers had normal pulmonary function. During the physical examination, wheezing was heard only in an asthma patient. No specific symptoms were observed in the other workers. Chest radiography revealed no specific findings in any of the patients. In terms of working history, with the exception of 1 worker who had asthma, 2 workers performed work for only 2–3 hours at the distance farther than 1 km from the accident site, which does not correspond to the place where and the time when they could have been exposed to high concentrations or large amounts of hydrogen fluoride. However, as only the workers who did not use respiratory protection experienced dyspnea, a more detailed study is required to further investigate the effects of the use of respiratory protective devices against exposure to hydrogen fluoride.

According to the pulmonary function test results in this study, the personnel who worked in close proximity to the accident site had significantly decreased FVC. According to one experimental study, when healthy male workers were exposed to low hydrogen fluoride levels while performing professional duties, exposure time increased with the decrease in FVC, consistent with the result of our study. Decreased FVC may lead to air trapping due to slight obstruction of the small airways in the lungs [15]. However, if respiratory symptoms due to the inhalation of hydrogen fluoride worsen, upper airway obstruction may occur as a result of laryngeal edema, laryngospasm, and bronchospasm [29,30]. When exposed to high concentrations up to 170 ppm, irritant-induced asthma develops, which results in obstructive ventilatory impairment [31]. Among the 31 participants (8.9%) in this study who had decreased FEV1 or FEV1/FVC, 1 subject with previous asthma symptoms had dyspnea accompanied with wheezing sounds, whereas the other workers exhibited no particular symptoms in the respiratory organs. Moreover, all the workers had no specific findings during the chest radiographic examination. In this study, no cases of serious upper airway obstruction or irritant-induced asthma due to inhalation of hydrogen fluoride were encountered. FEV1 or FEV1/FVC may be decreased owing to respiratory tract obstruction due to hydrogen fluoride, as well as to the proficiency of the patient in the pulmonary function test and the patient’s mood [3]. Therefore, further study on the relationship between hydrogen fluoride and obstructive ventilatory impairment is required.

If hydrogen fluoride is absorbed in the body, blood fluoride increases and bonds with body calcium and magnesium-forming insoluble salts, and may cause hypocalcemia, hypomagnesemia, and hyperkalemia [13,16,32]. Hypocalcemia may lead to convulsions and decreased myocardial contractile power, whereas hyperkalemia may cause ventricular fibrillation and other arrhythmias [29]. In addition, hydrogen fluoride suppresses enzymes in the Krebs cycle and Na/K^+^ ATPase pump cells, which may result in cell death and cellular energy failure [4]. However, in this study, none of the subjects had serum calcium concentrations outside of the reference range. No statistical differences were found in serum calcium concentration depending on the distance from the accident site, working time, use of protective devices. A total of 29 people had abnormal ECG findings, but determining their ECG results before they were exposed to hydrogen fluoride was impossible, thus the limitation of comparing between pre- and post-exposure ECG results being impossible. However, it was possible to confirm that there was no statistically significant difference in the result depending on the distance from accident site (p = 0.200), working time (p = 0.330), and use of protective devices (p = 0.230). Thus, both those who worked within a short distance and those who worked within a long distance from the accident site experienced upper airway irritation due to hydrogen fluoride inhalation, as well as symptoms in the lower airways and gastrointestinal system. However, no blood absorption levels that could lead to hypocalcemia or hyperkalemia were found.

In this study, it was impossible to use equipment for testing hydrogen fluoride levels; therefore, hydrogen fluoride levels in the air or urine fluoride levels, the biological exposure marker, were not measured. Instead, markers that indirectly reflected exposure level, such as distance to the accident site [7], the time of performing collection work, and individual use of respiratory protective devices, were analyzed. However, as SCBAs supply oxygen for approximately only 50 minutes, firefighters could not use them for an extended period [9]. In many cases, while they were waiting in the vicinity of the accident site, they did not use SCBAs but used other protective devices instead. Therefore, realistically and accurately classifying the use of protective devices was difficult. In addition, the use of chemical and all-body protection suits was not studied, and its relationship with skin symptoms was not investigated in the present study. Furthermore, as the participants of this study were hospitalized in October, the season during which flu and other upper airway infections are common, the possibility that some of the study subjects were hospitalized because of a common upper airway infection could not be excluded [7]. In addition, a mean duration of 12.5 days passed from performing collection to hospitalization (range, 3–22 days), indicating the possibility of symptom alleviation and recall bias. Moreover, this study had the limitation of being performed based only on those people who did collection work at the accident site and those who were hospitalized in only 1 hospital for evaluation for possible effects of hydrogen fluoride on health, which does not reflect all the workers who performed collection duties. However, literature on hydrogen fluoride mostly covers cases of skin and respiratory organ damage that resulted from workplace accidents. Studies on health hazard among firefighters who inhaled hydrogen fluoride are rare. This study has significance in that it investigated the health hazard among firefighters, police officers, and other personnel who performed collection duties at the hydrogen fluoride spill accident site.

The duties of a firefighter are characterized with the possibility of acquiring posttraumatic stress disorder (PTSD) due to exposure to fire in sites of disaster, death, inhalation of harmful gases, and exposure to infectious diseases. Secondary consequences of the firefighter’s work are decreased job performance, depression, alcoholism-related problems, and other mental health problems [33,34]. The potential risk for morbidity from PTSD and other stress-related diseases as consequences of the collection work was increased during the hydrogen fluoride spill. Therefore, active intervention is necessary, particularly by identifying the high-risk group and providing psychological counseling. Moreover, after a large-scale regional community hydrogen fluoride spill accident, a broad follow-up study should be conducted with the local residents. A previous study reported that approximately 20–40% of the subjects in a group with high exposure continued to experience respiratory organ, throat, and gastrointestinal symptoms 2 years after the accident [13]. Thus, additional continuous follow-up observation should be conducted among the firefighters, police officers, and other personnel who may be included in the high-risk group.

## Conclusions

This study investigated and analyzed the clinical characteristics and working history of firefighters who performed collection work at the accident site of the hydrogen fluoride spill that occurred on September 27, 2012, in a chemical plant in the National Industrial Complex in Gumi City. When the workers who performed collection duties at the accident site worked in close proximity to the accident site, performed the work for a long time, or had no respiratory protection, they experienced upper and lower airway, gastrointestinal tract, and neurological symptoms. It is necessary to continue follow-up observation among these workers to evaluate for stress-related diseases and physical symptoms.

### Consent

Written informed consent was obtained from the patient for the publication of this report and any accompanying images.

## Competing interests

The authors declare that they have no competing interests.

## Authors’ contributions

All authors had access to the data and played a role in writing this manuscript. SYC conceived and designed the study. SYY and JSK were involved in writing the manuscript. JYN and JHY performed the data collection. SYC and KHW performed the statistical analysis, the interpretation of data. YBK had critically revised the manuscript. All authors read and approved the final manuscript.
